# CD8^+^ T Cell Exhaustion in Cancer

**DOI:** 10.3389/fimmu.2021.715234

**Published:** 2021-07-20

**Authors:** Joseph S. Dolina, Natalija Van Braeckel-Budimir, Graham D. Thomas, Shahram Salek-Ardakani

**Affiliations:** Cancer Immunology Discovery, Pfizer, San Diego, CA, United States

**Keywords:** T cell exhaustion, PD-1/PD-L1, T cell trafficking, tumor immunity, cancer immunotherapy, CXCR3, co-stimulatory/inhibitory receptors, stem-like CD8^+^ T cells

## Abstract

A paradigm shift in the understanding of the exhausted CD8^+^ T cell (T_ex_) lineage is underway. Originally thought to be a uniform population that progressively loses effector function in response to persistent antigen, single-cell analysis has now revealed that CD8^+^ T_ex_ is composed of multiple interconnected subpopulations. The heterogeneity within the CD8^+^ T_ex_ lineage is comprised of immune checkpoint blockade (ICB) permissive and refractory subsets termed stem-like and terminally differentiated cells, respectively. These populations occupy distinct peripheral and intratumoral niches and are characterized by transcriptional processes that govern transitions between cell states. This review presents key findings in the field to construct an updated view of the spatial, transcriptional, and functional heterogeneity of anti-tumoral CD8^+^ T_ex_. These emerging insights broadly call for (re-)focusing cancer immunotherapies to center on the driver mechanism(s) underlying the CD8^+^ T_ex_ developmental continuum aimed at stabilizing functional subsets.

## Introduction

T cell exhaustion is a blanket term covering all of the dysfunctional states that exist within antigen-specific CD8^+^ T lymphocytes as first described in the framework of chronic viral infection, where these cells persist but are unsuccessful in clearing a pathogenic threat (1). Blockade of surface co-inhibitory receptors such as programmed death 1 (PD-1) expressed by CD8^+^ T_ex_ was shown to reinvigorate cytolytic cell-mediated immune responses leading to the eradication of some persistent viruses (2). Later found in cancer, CD8^+^ T_ex_ are found to be equally hyporesponsive to anti-tumor immunotherapies (3). Cells expressing PD-1 were thought to be rescued by ICB *via* simple unidirectional reversion from the unresponsive, exhausted state (2). In cancer, this was also believed to involve dysfunctional CD8^+^ T_ex_ expressing high levels of PD-1, primarily residing in the tumor microenvironment (TME) (3).

Recent advances in single-cell transcriptomics and genome-wide epigenetic profiling comparing normal tissue, peripheral blood, and the lymphoid compartment to tumor parenchyma have challenged this view. New insights have been made regarding the spatial arrangement and heterogeneity of CD8^+^ T_ex_ and their modulation by ICB (3). We now understand that PD-1 expression is not an absolute measure of cellular dysfunction and senescence. Instead, PD-1 intensity reflects a complex heterogeneity existing within CD8^+^ T_ex_ (4). Emergent data now casts CD8^+^ T_ex_ as a developmental continuum, where the lineage is comprised of stem-like PD-1^lo^CD8^+^ T_ex_ precursors/progenitors that ultimately give rise to terminally dysfunctional PD-1^hi^CD8^+^ T_ex_ (3). In cancer, these CD8^+^ T_ex_ subsets appear to be unevenly spread amongst normal peripheral versus tumoral tissues and are differentially responsive to ICB (3).

This review discusses the original works that first identified CD8^+^ T_ex_ and more contemporary reports describing this population as a developmentally distinct lineage using chronic viral infection. We draw on these data as a basis to further our understanding of CD8^+^ T_ex_ function during anti-tumor immune responses and elucidate the cellular dynamics and molecular pathways underlying the success and limitations of ICB. Throughout this review, we highlight fundamental knowledge gaps regarding the factors underlying control over CD8^+^ T_ex_ heterogeneity.

## Translating CD8^+^ T Cell Exhaustion From Chronic Infection to Cancer: A Common Role of Persistent Antigen

**Figure 1:**
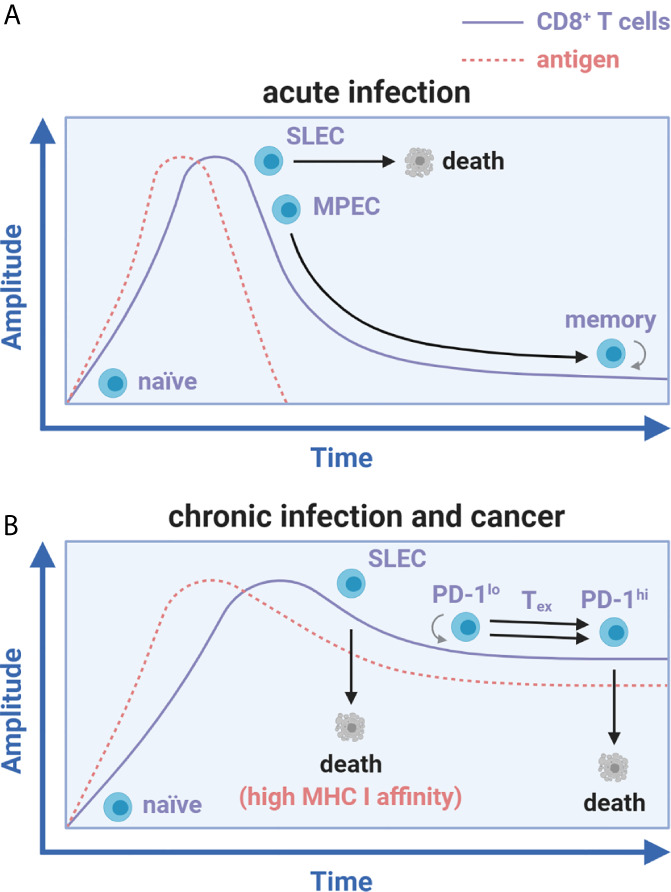
Antigen load differentially influences CD8^+^ T cell memory and exhaustion fates. CD8^+^ T cell differentiation during acute infection versus chronic infection and cancer. **(A)** Activation of naïve CD8^+^ T cells during acute infection leads to SLEC and MPEC differentiation. Upon antigen clearance, SLECs undergo apoptosis while MPECs survive and differentiate into long-lived, self-renewing memory CD8^+^ T cells. **(B)** With chronic infection and cancer, SLEC specific to peptides of high MHC I affinity develop and prematurely die while the MPEC subset does not form. Instead of memory formation, CD8^+^ T cells against peptides of low MHC I affinity expand, exhaust (in a unidirectional PD-1^lo^ to PD-1^hi^ transition), and die in a continued stalemate against persistent antigen.

The origin of the term T cell exhaustion goes back to the notable decay of T cell responses first documented in human immunodeficiency virus (HIV)-infected patients (5). It was speculated that viral persistence was linked with loss of function observed in these declining T cell subsets. CD8^+^ T cell functionality (the ability to rapidly expand after priming, produce effector cytokines and cytolytic molecules, and contract to form memory) characterizes acute recognition of cognate antigen during vaccination or natural, but eventually cleared, viral/bacterial infections (6, 7). Throughout the expansion phase, naïve CD8^+^ T cells differentiate into short-lived effector cells (SLEC) or memory precursor effector cells (MPEC) (6). Upon contraction and antigen clearance, most SLECs die while MPECs survive to form memory CD8^+^ T cells for long-term protective immunity (**Figure 1A**) (6, 8). The existence of a CD8^+^ T_ex_ counterpart to the conventional acute immune response was formally realized at the height of the HIV pandemic when Zinkernagel et al. exposed mice to acute (Armstrong and WE) versus chronic (Clone 13 and DOCILE) strains of lymphocytic choriomeningitis virus (LCMV), a rodent-borne negative-stranded RNA arenavirus (9). In this seminal work, Clone 13 and DOCILE strains persisted in infected mice for greater than 200 days at high inocula while transferred T cell receptor (TCR) transgenic virus-specific CD8^+^ T cells disappeared or crashed without contraction to memory (9). Initial exposure of select viral strains and doses thus appeared to scale cellular immunity towards protection or completely ‘exhausted’ the response, as it was coined.

This finding was later examined by two teams [Zajac, Wherry, and Ahmed et al. (10, 11) along with Gallimore and Rammensee et al. (12)] concurrent with the advent of major histocompatibility complex class I (MHC I) tetramer staining technology to track endogenous antigen-specific CD8^+^ T cells. It was found that initially dominant cytolytic CD8^+^ T cell responses against LCMV-derived peptides with high MHC I affinity (NP_396-404_ and GP_34-42_) were rapidly deleted, just as Zinkernagel initially observed (9). However, functionally inadequate responses against low/moderate affinity peptides (GP_33-41_ and GP_276-286_) persisted for greater than 60 days post-infection (**Figure 1B**) (10–12). These results showed that constantly elevated viral load and peptide affinity for MHC I strongly correlated with the degree of exhaustion and determined deletion versus persistence of CD8^+^ T_ex_ (10, 11). Low avidity persisting cells exhibited a hierarchical loss of functionality at relatively low viral loads, which manifested as a dramatic decrease in proliferation, cytotoxicity, and cytokine production (2, 10, 11). Interleukin-2 (IL-2) and tumor necrosis factor (TNF) were lost early, whereas interferon-γ (IFN-γ) production persisted longer after infection (2, 10, 11). At elevated viral doses or with depletion of CD4^+^ T cell help, these gradual losses of functionality (or dysfunction) resulted in a nearly complete reduction in effector function followed by cell death/deletion (9–11). This process translated to HIV infection and other chronic or latent viral infections in humans, including hepatitis B and C viruses (HBV/HCV), herpes simplex virus (HSV), cytomegalovirus (CMV), human papillomaviruses (HPV), Epstein-Barr virus (EBV), and others (2, 13).

**Figure 2:**
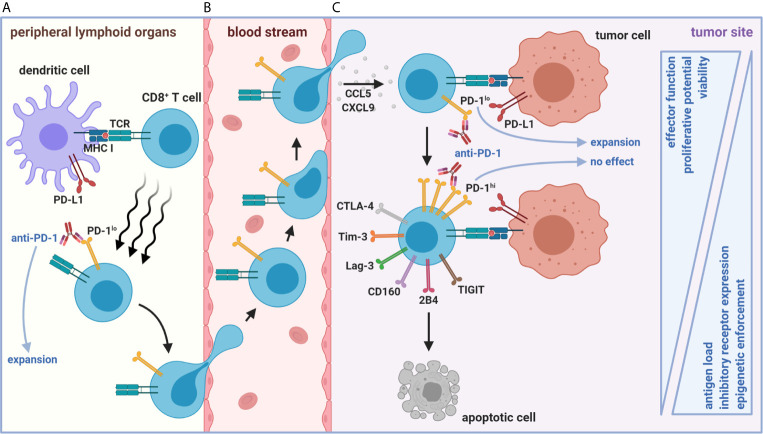
Spatiotemporal organization of early versus late stages of tumor-mediated CD8^+^ T cell dysfunction. **(A)** Naïve CD8^+^ T cell priming against tumor antigen in peripheral LNs (or intratumoral TLS, not depicted) results in the formation of a stem-like PD-1^lo^CD8^+^ T cell population with self-renewing properties. **(B)** This population represents an active reservoir of cells that can give rise to effector-like PD-1^lo^CD8^+^ T_ex_ after chemokine-mediated trafficking to and positioning within the TME via CCL5 and CXCL9. **(C)** However, persistent antigen load in the TME eventually forces continued differentiation of these cells into terminally dysfunctional PD-1^hi^CD8^+^ T_ex_. The PD-1^hi^ state is accompanied by heightened co-inhibitory receptor expression (including Tim-3, Lag-3, CD160, 2B4, TIGIT, and CTLA-4) and progressive loss of effector functions. Once CD8^+^ T_ex_ enter a PD-1^hi^ state, epigenetic enforcement prevents de-differentiation back to functional stem-like and effector-like PD-1^lo^ states. Anti-tumoral responses facilitated by ICB (*e.g.*, anti-PD-1) arise from expansion from only lymphoid or intratumoral PD-1^lo^CD8^+^ T_ex_ subsets. The functionally inferior, ICB-resistant PD-1^hi^CD8^+^ T_ex_ fate ultimately culminates in apoptosis.

**Figure 3:**
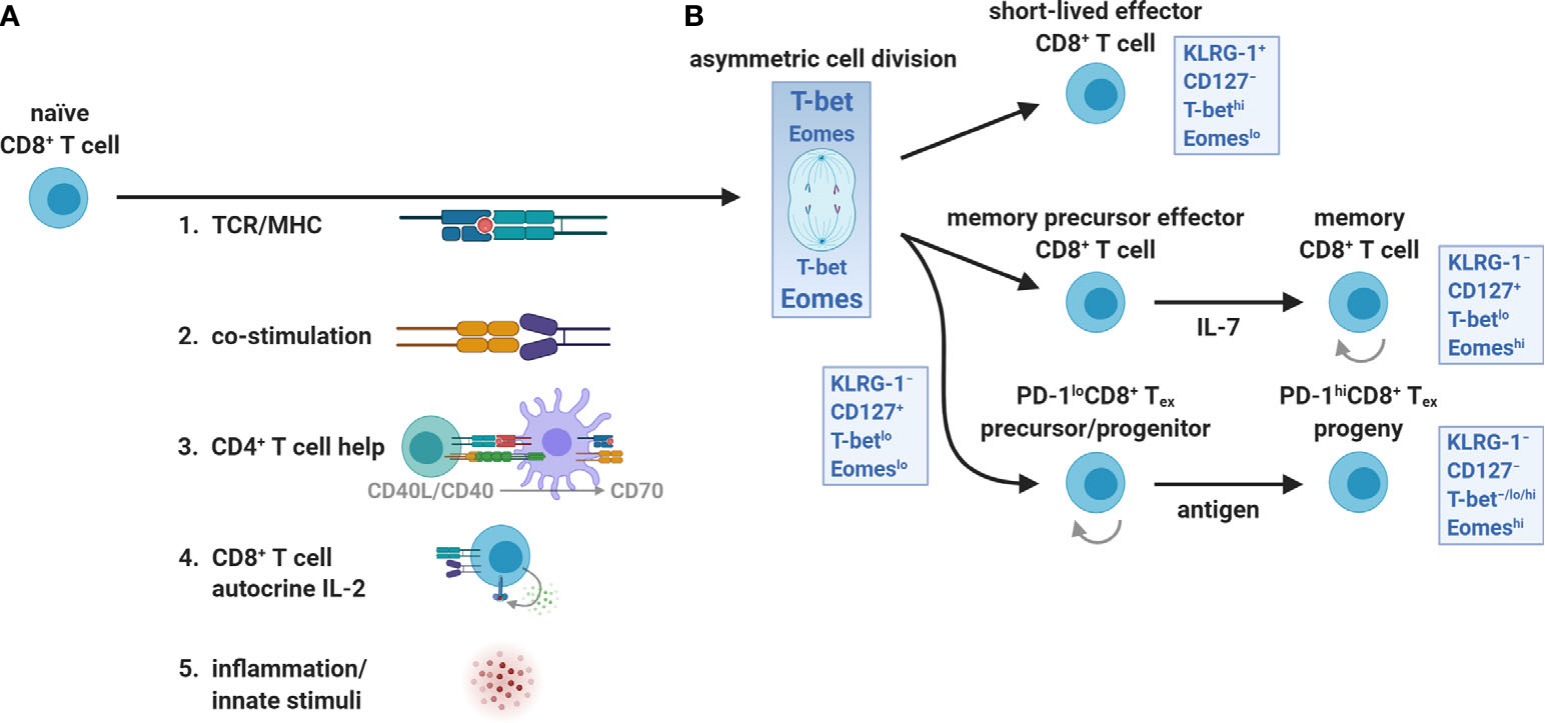
T-bet and Eomes partitioning during CD8^+^ T cell priming and expansion. **(A)** The orientation and strength of TCR/MHC ligation, co-stimulation (*e.g.*, CD28 interaction with CD80 and CD86), CD4^+^ T cell help (CD40L/CD40 licensing of DCs including up-regulation of MHC I, CD80/CD86, CD70, and third signal cytokines), autocrine IL-2 exposure, and innate inflammatory stimuli (danger- and pathogen-associated molecular patterns) all influence the activation, survival, and differentiation of naïve CD8^+^ T cells. **(B)** CD8^+^ T cells integrate these input events at priming and during the first division. The uneven partitioning of T-bet and Eomes favors SLEC (effector) versus MPEC (memory) differentiation early after activation, respectively. In contrast, the CD8^+^ T_ex_ lineage requires both transcription factors and retains some features of memory cells including self-renewal of PD-1^lo^ subsets and expression of memory-associated transcription factors and survival molecules. The reliance on homeostatic cytokines (predominantly IL-7) versus persistent antigen for development and self-renewal distinguishes memory from PD-1^lo/hi^ exhaustion lineages, respectively.

A common feature of chronic viral infection and cancer is that both are prolonged diseases characterized by an overt persistence of antigen (4). CD8^+^ tumor-infiltrating lymphocytes (TILs) are similarly hyporesponsive as those found during chronic viral infection but are instead caught in an *in vivo* détente against the progressively growing tumor (14). Patient TILs are also tumor antigen-specific and MHC-restricted, supporting the role of chronic antigen persistence in driving T cell exhaustion (15, 16). Importantly, antigen displayed in the TME appears to fully drive CD8^+^ TIL exhaustion towards completion, whereas the periphery does not, as shown in preclinical models (17, 18). These data imply that the periphery may be an active reservoir of functional precursors to CD8^+^ T_ex_ (**Figures 2A, B**) before the physical invasion of tumors and chronic exposure to tumor-derived antigen (**Figure 2C**)—a spatial feature distinct from Clone 13 infection. Although persistent antigen plays a significant role in sustaining CD8^+^ T_ex_ for terminal differentiation in the tumor, other early events in CD8^+^ T cell activation may also be critical for the initial programming of exhaustion in the periphery or specialized tumor niches, including TCR signal quality/strength (NFAT versus NFAT/AP-1 signaling, discussed below), co-stimulation, IL-2 availability (with associated CD4^+^ T cell helper signals), and inflammatory cues in the first few divisions (**Figure 3A**) (1, 10, 19–22).

Contemporary studies comparing chronic viral infection to cancer have sought to identify common CD8^+^ T_ex_ transcriptional signatures. At first glance, both tumor- and chronic virus-specific CD8^+^ T cells possess significant enrichment of genes related to recent TCR signaling (*Batf*, *Egr2*, *Ezh2*, *Irf4*, *Nfatc1*, *Nfatc2*, *Nr4a1*, *Nr4a2*, and *Nr4a3*) (17, 18, 23–25). This observation reinforces that constant engagement of persistent antigen is a dominant driver of exhaustion. These dominant transcriptional features are notably shared in a direct comparison of CD8^+^ T_ex_ isolated from HIV-infected and melanoma patients. They can also be recapitulated in CD8^+^ T cells given repeated cognate peptide stimulations *in vitro* (26, 27). However, significant disparities in CD8^+^ T_ex_ transcriptional phenotypes also exist between cancer and viral settings. These appear to be unrelated to exhaustion *per se*, where TIL uniquely retain gene ontologies associated with the suppressive TME and are devoid of pathways linked with virally-induced inflammation (3, 18, 28).

## Reversing T Cell Exhaustion: Lessons Learned From Immune Checkpoint Blockade

The onset of exhaustion coincides with the surface expression of co-inhibitory receptors, which control CD8^+^ T cell function (2). It has been considered that these immune checkpoints, which include PD-1 (among others), evolved to constrain T cell activation, preventing excessive adverse inflammatory and autoimmune events (29, 30). They also seem to function throughout exhaustion and not merely correlate with loss-of-function, as blocking interactions between PD-1 and its ligand (PD-L1) can restore the function and survival of CD8^+^ T_ex_ (2, 31). With Clone 13 infection, ICB of PD-(L)1 was initially shown by Barber and Ahmed et al. to reinvigorate CD8^+^ T_ex_ (31). Importantly, restoration of the response originated from PD-1^+^CD8^+^ T_ex_ and not from *de novo* naïve PD-1^−^CD8^+^ T cell priming (31). This early study led to the idea that reinvigoration of CD8^+^ T_ex_ was practically synonymous with ‘reversal’ of exhaustion. At this time, ICB was rapidly advanced into the clinic and established a new paradigm for cancer treatment, leading to durable responses in a limited set of patients (32, 33). Despite its early success and first recordings of tails in long-term endpoint survival curves, the mechanism of action behind ICB remained elusive.

Blackburn and Wherry et al. uncovered an underlying complexity within the presumed homogenous PD-1^+^CD8^+^ T_ex_, where this population could be further separated into PD-1^lo^ and PD-1^hi^ subsets (34). A hypothesis emerged from this that proposed PD-1^lo^ cells differentiate into the PD-1^hi^ subset as CD8^+^ T cells exhaust. Inherent in this theory, reinvigoration did not equate to the reversal of exhaustion (herein defined as a PD-1^hi^ to PD-1^lo/−^ transition). Beneficial responses rather arose solely from the mobilization of less exhausted, permissive PD-1^lo^ cells instead of PD-1^hi^ terminally exhausted counterparts. After transferring day 30 Clone 13-generated PD-1^lo^ and PD-1^hi^ sorted cells into naïve mice subsequently re-challenged with Clone 13 in the presence or absence of anti-PD-L1, Blackburn and Wherry et al. showed that only PD-1^lo^CD8^+^ T_ex_ could proliferate in response to ICB (34). Similar transfer experiments also revealed that PD-1^lo^CD8^+^ T_ex_ were more effective at controlling viral load and remained less apoptotic compared to PD-1^hi^CD8^+^ T_ex_ (34). The PD-1^hi^ subset was later associated with expression of additional co-inhibitory receptors, including T cell immunoglobulin domain and mucin domain protein 3 (Tim-3), lymphocyte activation gene 3 (Lag-3), natural killer cell receptor BY55 (CD160), signaling lymphocytic activation molecule 4 (2B4), cytotoxic T-lymphocyte-associated protein 4 (CTLA-4), and T cell immunoreceptor with immunoglobulin and ITIM domains (TIGIT), where cells having heightened co-expression appeared more exhausted (**Figure 2C**) (24, 35–38).

## Dawn of Stem-Like Precursors and Progenitors of Exhausted CD8^+^ T Cells

**Figure 4:**
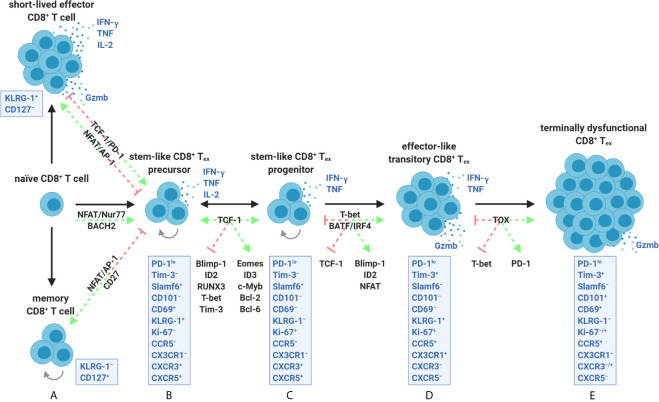
The CD8^+^ T cell exhaustion lineage is comprised of a continuum of transcriptionally and epigenetically controlled states. **(A)** Activation of naïve CD8^+^ T cells for SLEC and MPEC/memory differentiation is optimally driven by transcription factors such as NFAT/AP-1 and sufficient co-stimulation (*e.g.*, CD27). Early development of stem-like CD8^+^ T_ex_ precursors instead involves partnerless NFAT, lack of co-stimulation/help, constant Nur77 activity, and/or strict dependence on BACH2. These events appear to be stabilized by TCF-1 activity and PD-1 dampening of chronic TCR ligation. **(B, C)** TCF-1 further supports stemness (ability to survive, self-renew, and proliferate) by promoting Eomes, ID3, c-Myb, Bcl-2, and Bcl-6 expression while antagonizing effector-associated transcription factors including Blimp-1, ID2, RUNX3, and T-bet. Stem-like CD8^+^ T_ex_ precursors **(B)** and progenitors **(C)** are collectively marked by a PD-1^lo^Tim-3^−^Slamf6^+^ surface profile and varied expression of CXCR3 and CXCR5 in specific tumor settings. Although equally stabilized by TCF-1, precursors can be distinguished from progenitors as being quiescent, LN-resident, less reliant on antigen, and having a CD69^+^KLRG-1^+^Ki-67^−^ profile. **(C, D)** TCF-1 down-regulation coupled with ongoing exposure to persistent antigen drives constant BATF and IRF4 signaling (which positively feedback on partnerless NFAT activity) and T-bet expression. T-bet additionally overrides a TCF-1 memory-like program by supporting Blimp-1 and ID2 activity leading to an effector-like transitory PD-1^lo^Tim-3^+^ state marked by initiation of granzyme B production. **(D, E)** Continued NFAT activity ultimately leads to TOX upregulation within this subpopulation, which epigenetically enforces terminal exhaustion, inhibits T-bet-mediated effector programming, and promotes heightened PD-1 expression. Transitory cells **(D)** are discriminated from terminally dysfunctional cells **(E)** by a PD-1^lo^Tim-3^+^CD101^−^KLRG-1^+^CX3CR1^+^ surface phenotype, remnant IFN-γ and TNF production, and having high proliferative potential. Terminally dysfunctional PD-1^hi^Tim-3^+^CD8^+^ T_ex_ co-express multiple co-inhibitory receptors (not depicted), cannot proliferate, have diminished polyfunctionality, but retain granzyme-based cytolytic potential. PD-1^lo^ precursors, progenitors, and transitory CD8^+^ T_ex_ subpopulations are amenable to ICB **(B–D)**, whereas terminally dysfunctional PD-1^hi^CD8^+^ T_ex_ are not **(E)**. Precursors and progenitors may interconvert, whereas differentiation into transitory and terminally dysfunctional subsets is unidirectional.

It also became apparent that heterogeneity existed at a deeper level than surface PD-1, where CD8^+^ T_ex_ appeared to use the same T-box family transcription factors, T-box expressed in T cells (T-bet) and eomesodermin (Eomes), for SLEC and MPEC lineage commitment, respectively, but with different expression patterns, nuclear localization, and developmental connectivity (4, 39–41). In response to TCR/MHC ligation and orientation of the immune synapse, a naïve CD8^+^ T cell will asymmetrically divide and unequally partition T-bet and Eomes, separately dictating effector versus memory fates from the first division (22, 42–45). Distinct from SLECs and MPECs, T-bet and Eomes were shown to be dually required for CD8^+^ T_ex_ development (41). In addition, these transcription factors appeared to arise at different stages of CD8^+^ T_ex_, where PD-1^lo^T-bet^lo^CD8^+^ T_ex_ were found to increase Eomes expression and sustain its nuclear localization, divide, and differentiate into PD-1^hi^T-bet^−/lo/hi^Eomes^hi^CD8^+^ T_ex_ (**Figures 3B** and **4A**) (40, 41, 46). This differential usage of T-bet and Eomes also suggested that CD8^+^ T_ex_ was a distinct lineage.

Ahmed et al., therefore, reexamined the Clone 13 model and the underlying CD8^+^ T_ex_ transcriptional heterogeneity at the core of the PD-1^lo/hi^ dichotomy (47). Transcriptional analyses of the PD-1^lo^ population revealed an association with *Icos* (inducible T cell co-stimulator; ICOS), *Cxcr5* (C-X-C motif chemokine receptor 5; CXCR5), *Bcl6* (B cell lymphoma 6; Bcl-6), and *Tcf7* (T cell factor 1; TCF-1) expression reminiscent of CD4^+^ T follicular helper cells (T_fh_), which is why CD8^+^ T_ex_ are sometimes referred to as T_fh_-like (47, 48). TCF-1 acts as the main transcription factor downstream of Notch receptors as part of the evolutionarily conserved Wnt signaling pathway, known to be critical for T cell thymic development and memory formation (49). TCF-1, together with forkhead box protein O1 (FOXO1), promotes stemness in CD8^+^ T cells by inhibiting expression of effector-associated genes including *Prdm1* (B lymphocyte-induced maturation protein-1; Blimp-1), *Runx3* (Runt-related transcription factor 3; RUNX3), *Id2* (inhibitor of DNA binding 2; ID2) and *Tbx21* (T-bet) and favoring central memory by promoting *Eomes* and *Bcl6* expression (50, 51). Blimp-1, in particular, is known to act as a rheostat balancing the promotion of cytolytic granzyme B production and terminal dysfunction in CD8^+^ T_ex_—events directly countered by TCF-1 (**Figures 4B, C**) (49, 51, 52). Other associations of PD-1^lo^CD8^+^ T_ex_ with the high affinity IL-7 receptor chain (IL-7Rα), L-selectin (CD62L), and mitochondrial β-oxidation (fatty acid metabolism) pathway enrichment suggested shared common features with self-renewing CD8^+^ T memory precursors (47). Moreover, PD-1^hi^Tim-3^+^CD8^+^ T_ex_ did not produce effector cytokines but did retain cytolytic *Gzma* (granzyme A), *Gzmb* (granzyme B), and *Prf1* (perforin) expression (47, 53, 54). Sorting and transferring PD-1^lo/hi^ subsets into infection-matched hosts based upon CXCR5 positivity validated that PD-1^lo^Tim-3^−^CXCR5^+^CD8^+^ T_ex_ marked a self-renewing population that gave rise to PD-1^hi^Tim-3^+^CXCR5^−^CD8^+^ T_ex_ (47). Further and more critical, anti-PD-L1 blockade triggered a proliferative burst only within the stem-like PD-1^lo^Tim-3^−^ subset and facilitated transitions to the treatment-refractory PD-1^hi^Tim-3^+^ fate (47).

Since TCF-1 expression was generally known to maintain stemness in hematopoietic stem cells, its role in the PD-1^lo/hi^ T_ex_ progenitor/progeny relationship was determined (47). In *Tcf7* ^−/−^ mice, PD-1^lo^CD8^+^ T_ex_ fail to develop and cannot seed the exhaustion lineage (47). In contrast, transgenic overexpression of *Tcf7* was found to stabilize PD-1^lo^ stem-like cells and lead to more durable CD8^+^ T cell responses during Clone 13 infection and within the B16-GP_33-41_ melanoma models, implicating TCF-1 as a critical factor for the inception of T_ex_ (55). TCF-1 was later shown to support the expression of *Id3* (ID3), *Eomes*, *Myb* (transcriptional activator Myb; c-Myb), and *Bcl2* (Bcl-2), allowing PD-1^lo^CD8^+^ T_ex_ to survive negative downstream signals from PD-1 early after priming (**Figures 4B, C**) (56, 57).

The factors governing the expression of TCF-1 within stem-like PD-1^lo^CD8^+^ T_ex_ have only recently been investigated. During chronic DOCILE infection of mice, the amount of antigen but not inflammation rapidly promotes the formation of the TCF-1^+^ population (57). Inconsistent with the need for chronic antigen during its establishment, some elements of the exhaustion program (maintenance of a PD-1^hi^ dysfunctional profile) were paradoxically shown to be stable after CD8^+^ T_ex_ transfer to antigen-free conditions (58). This suggests that the CD8^+^ T_ex_ lineage has some component(s) shared with memory CD8^+^ T cells, including slow homeostatic self-renewal by IL-7 and IL-15 (4, 58). In support of this, GP_33-41_-specific CD8^+^ T cells deficient in BACH2 (a transcription factor that promotes memory cell development by limiting TCR-mediated transcriptional changes) fail to form any stem-like PD-1^lo^TCF-1^+^CD8^+^ T_ex_ (57, 59). Conversely, the progression of the stem/memory-like PD-1^lo^TCF-1^+^ state to the TCF-1^−^PD-1^hi^ terminally exhausted fate is halted by deleting BATF and IRF4 (two transcription factors linked with constant TCR signaling and known to destabilize TCF-1) (**Figures 4C, D**) (25, 57). Therefore, T cell-intrinsic TCF-1 expression appears to rely on a low but brief TCR signaling threshold compromised by ongoing antigenic exposure.

Other studies have oppositely shown that PD-1^hi^CD8^+^ T_ex_ generated from Clone 13 infection are less stable without antigen, where these cells inevitably decline and cannot mount a recall response (21, 60). Discrepancies regarding CD8^+^ T_ex_ stability in the presence/absence of antigen may be due to the frequency and quality of TCF-1^+^ stem-like cells at hand. A unified atlas of 12 studies spanning cancer and chronic viral infection has recently revealed that bifurcation of memory commitment from a dysfunctional program occurs early (in less than 7 days following antigen encounter) (61). With preclinical cancer models, the time of initial antigen encounter is less controlled for compared to viral infection. Nevertheless, it has been shown that PD-1^lo^CD8^+^ TIL removed early after tumor injection (likely containing an increased frequency of TCF-1^+^ cells) followed by transfer into naïve hosts and infection with *Listeria monocytogenes* 3-4 weeks later can mount a memory response whereas fully exhausted PD-1^hi^CD8^+^ TIL isolated at later time points cannot (18). In addition, stem-like PD-1^lo^TCF-1^+^CD8^+^ T_ex_ can be divided into CD69^+^Ki-67^−^ precursor cell and CD69^−^Ki-67^+^ progenitor cell subsets (and are thus differentiated as such in this review) (**Figures 4B, C**) (46). Precursors are lymph node (LN)-resident, speculated to depend less on antigen for a low baseline level of proliferation, and remain quiescent compared to a circulating progenitor pool (46). In healthy human subjects, TCF-1^+^ precursors specific to common chronic diseases such as latent EBV and CMV were shown to be present in the periphery and co-express PD-1, TIGIT, and granzyme K (62). These precursors are also embedded within steady-state stem-like/central memory CD8^+^ T cell populations traditionally defined as CCR7^+^CD45RO^+/−^CD95^+^ (62). Yet, no known mediator has been identified to date which controls functional memory versus stem-like PD-1^lo^CD8^+^ T_ex_ precursor differentiation (**Figures 4A, B**) (62). Precursors and progenitors have also been documented to reside in TIL fractions of murine B16 tumors and human melanoma (46). However, it remains to be determined if these small populations are biased in tumor versus LN organization and if CD69 positivity/negativity within the bulk TCF-1^+^PD-1^lo^ population determines true stemness and reactivity to ICB and/or antigen.

## Transcriptional and Epigenetic Events Critical for the Establishment of Terminal Exhaustion

Complementing these approaches, total CD8^+^ T_ex_ were shown to possess a fixed chromatin state distinct from effector and memory cells by ~6,000 open chroman regions before or after exposure to anti-PD-L1 (21, 63, 64). This reinforces that terminal CD8^+^ T_ex_ represents a distinct lineage unable to differentiate into *bona fide* memory cells. Second, ICB-mobilized stem/effector-like PD-1^lo^ populations themselves exhaust and eventually mirror pre-treatment PD-1^hi^CD8^+^ T_ex_. The unique epigenetic signature of CD8^+^ T_ex_ in Clone 13-infected mice was also shown to be conserved in HIV-infected and melanoma patients (26, 63). Although both acutely activated CD8^+^ T cells and CD8^+^ T_ex_ generally express PD-1, assay for transposase-accessible chromatin sequencing (ATAC-Seq) distinguishes these populations, with CD8^+^ T_ex_ possessing many unique features, including *de novo* accessibility of the region at −22.4 kb upstream of the murine *Pdcd1* (PD-1) locus containing a *Nr4a1* (Nur77) binding motif (17, 63).

Downstream from TCF-1-mediated subsistence of PD-1^lo^CD8^+^ T_ex_, thymocyte selection-associated high-mobility group (HMG) box protein, TOX, becomes co-upregulated alongside PD-1 and is associated with the epigenetic signatures demarcating terminal lineage commitment within PD-1^hi^CD8^+^ T_ex_ (24, 56, 65–67). TOX is a nuclear protein that binds DNA in a structure-dependent manner (not sequence-dependent) (64). TOX directly interacts with histone acetyltransferase binding to ORC1 (HBO1) and indirectly coordinates activity with DNA methyltransferases 3A (DNMT3A), 3B (DNMT3B), and enhancer of zeste homolog 2 (EZH2) to epigenetically fix CD8^+^ T_ex_ towards terminal exhaustion (64). Ectopic TOX expression is sufficient to induce a full exhaustion transcriptional program in effector CD8^+^ T cells *in vitro* (65). In contrast, deletion of *Tox* in CD8^+^ TIL prevents exhaustion *via* decreased chromatin accessibility and expression of *Pdcd1*, *Havcr2* (Tim-3), *Cd244* (2B4), and *Tigit* (TIGIT) in the SV40-Tag-driven autochthonous liver cancer model (65). In the Clone 13 system, *Tcf7^flox/flox^Cd4^cre^* and *Tox^flox/flox^Cd4^cre^* mice (lacking TCF-1 and TOX in all T cells, respectively) results in favored development of effector-like KLRG-1^+^CD8^+^ T cells over the formation of PD-1^hi^CD8^+^ T_ex_ (**Figures 4A, D**) (24, 57, 68).

Recent findings by Ahmed (69) and Wherry (46) jointly demonstrate that stem-like cells are initially stable during Clone 13 infection. However, upon ICB treatment, these cells rapidly enter a T-bet-driven effector-like transitory state marked as CX3CR1^+^KLRG-1^+^CD101^−^PD-1^lo^Tim-3^+^ (**Figure 4D**), which rapidly proliferate, temporarily produce granzyme B, and eventually digress to fully exhausted CX3CR1^−^KLRG-1^−^CD101^+^PD-1^hi^Tim-3^+^CD8^+^ T_ex_ (**Figure 4E**) (46, 69). CD101 is not expressed at baseline in CD8^+^ T cells from healthy humans (70). Conversely, terminally differentiated CD101^+^PD-1^hi^CD8^+^ T_ex_ have recently been observed to correlate negatively with tumor grade and regional LN metastasis within epithelial ovarian cancer patients (70). Transcriptional analyses of murine and human TIL corroborate these results linking changes in naïve-like PD-1^−^Tim-3^−^CD8^+^ TIL before and after ICB (71). ICB appears to bifurcate PD-1^−^Tim-3^−^CD8^+^ TIL into a self-renewing stem-like state (expressing *Tcf7*, *Lef1*, and *Sell*) and an effector-like program (expressing *Klrg1*, *Cx3cr1*, *Slamf7*, and *Ifng*) farther downstream along a developmental trajectory to full exhaustion (71). These phenotypic changes (stem-like > effector-like transitory > terminal exhaustion) coincide with chromatin accessibility shifts controlled by multiple transcription factors including NFAT, Nur77, BATF, IRF4, TCF-1, T-bet, and TOX that appear to be coordinated with PD-1-mediated TCR dampening (**Figures 4A–E**) (25, 46, 69, 72). In other words, CD8^+^ T_ex_ seem to represent a lineage with limited differentiation capacity, existing within a series of fixed sequential epigenetic landscapes. Although reinvigoration of PD-1^lo^CD8^+^ T_ex_ can result in a detectable wave of transcriptionally ‘re-wired’ effector-like activity, the cells appear to be limited because they eventually exhaust in response to ICB and are unable to de-differentiate into *bona fide* effector or memory cells present during acute infection (21, 46, 63). In the context of tumor immunity, understanding where these transitions occur *in vivo* (LN versus TME) and how to stabilize the transitory effector-like state is key to maximizing the cytolytic potential of stem-like CD8^+^ T_ex_.

What governs late-stage cell fate decisions of stem-like PD-1^lo^CD8^+^ T_ex_ progenitors to commit to a terminally exhausted PD-1^hi^CD8^+^ T_ex_ fate is partially clear at best. Constant TCR signaling is likely involved as enforced nuclear factor of activated T cells (NFAT) activity in antigen-specific CD8^+^ T cells directly leads to *Tox* transcription (20, 24). Conversely, lack of *Nfatc1* (NFAT2) phenocopies loss of TOX (24). Further, TCR-responsive transcription factors, including BATF and IRF4, appear to positively feedback on *Nfatc1* transcription promoting PD-1^hi^Tim-3^+^ T_ex_ development (25). In contrast to NFAT1, NFAT2 itself is also known to favor the development of MPECs over SLECs (73). Imbalanced NFAT1 versus NFAT2 may also relate to skewing early T-bet and Eomes segregation in a primed CD8^+^ T cell to seed TCF-1^+^ stem-like progenitors even before ongoing direct downstream effects on TOX, and other exhaustion-associated genes are enforced. At a higher level, the overall balance between NFAT and CD28/AP-1 activity upon original and/or continued antigen encounter may be critical as anergic CD8^+^ T cells and CD8^+^ T cells primed in the absence of CD4^+^ T cell help or co-stimulation mirror many of the major transcriptional and epigenetic events that occur in PD-1^lo/hi^CD8^+^ T_ex_ in both chronic viral infection and cancer (19, 20, 26, 66, 74–80). Exposure to microenvironmental stressors (low glucose, high lipid) in the TME may also orchestrate the TOX-centric epigenetic program that characterizes the PD-1^hi^ dysfunctional phenotype by disrupting metabolic/mitochondrial fitness (81–83). Mitochondria tend to produce elevated amounts of reactive oxygen species (ROS) in CD8^+^ T_ex_, which was shown to facilitate nuclear entry of NFAT downstream of a Ca^++^ flux in both CD4^+^ and CD8^+^ T cells (81–84). How constant PD-1 signaling, TCR engagement, and altered metabolism control the transition from a TCF-1^+^ to TOX^+^ state *via* constant NFAT activity in CD8^+^ T_ex_ is an area where current knowledge is limited and is only starting to be investigated.

## Therapeutic Potential of CD8^+^ T Cell Progenitors in Cancer

The significance of stem-like TCF-1^+^PD-1^lo^CD8^+^ T_ex_ in governing ICB outcomes may lie in their pre-treatment frequency and crosstalk between other immune cell types during cancer. Surveys of TIL heterogeneity using single-cell RNA sequencing (scRNA-Seq) have indicated that activated, expanded, and exhausted CD8^+^ T cell subsets are variably present in different tumor samples and effectively cluster based on *Tcf7* expression (53, 85). For instance, Sade-Feldman et al. profiled 48 metastatic melanoma tumor biopsies, comprising 17 responder and 31 non-responder patients receiving ICB (85). scRNA-Seq phenotyping of CD8^+^ T cell clusters identified 6 clusters that were putatively annotated as belonging to early-activated, memory, effector, and exhausted lineages based upon cell surface marker expression profiles (85). All CD8^+^ T cell populations were observed in most patients, albeit to differing degrees (85). However, the relative frequency of intratumoral *Tcf7* ^hi^ versus *Tcf7* ^lo^ TIL clusters was predictive of patient responsiveness to ICB (85). It has since then been confirmed in preclinical models that small populations of stem-like PD-1^lo^Slamf6^+^TCF-1^+^CD8^+^ T_ex_ (with Slamf6 being a surrogate for TCF-1) and PD-1^hi^TOX^+^CD8^+^ T_ex_ indeed exist in the TME (86). In murine B16 melanoma, TILs retained some features of the epigenetic profile seen in CD8^+^ T_ex_ following Clone 13 infection, and anti-PD-1 treatment specifically drove stem-like PD-1^lo^ TILs to divide and convert into terminally exhausted PD-1^hi^ T_ex_ (86). In humans, stem-like TCF-1^+^CD8^+^ T_ex_ progenitors and terminally exhausted TCF-1^−^CD8^+^ T_ex_ have similarly been observed in multiple tumor indications (71, 86, 87).

As noted, Ahmed initially found that stem-like PD-1^lo^CD8^+^ T_ex_ express CXCR5; however, these cells co-express high amounts of *Ccr7* transcripts, migrate in response to a CCL19/21 gradient *in vitro*, and localize to the splenic T cell zone *in vivo* after Clone 13 infection (47). In this system, CXCR5 is expressed by both stem-like CD69^+^Ki-67^−^ precursors and CD69^−^Ki-67^+^ progenitors (46). The function of CXCR5 is less well known in cancer immunology but may relate to stem-like CD8^+^ T_ex_ positioning. Stem-like PD-1^lo^CD8^+^ TILs have been found to sporadically express CXCR5 depending on the tumor type (86, 88). In murine and human melanomas, CXCR5 positivity has thus far not tracked with stem-like PD-1^lo^CD8^+^ TIL (86). In contrast, CXCR5^+^ TILs can be found in non-small-cell lung carcinoma (NSCLC) tumors and may uniquely associate with intratumoral tertiary lymphoid structures (TLS) (88). More work is needed to understand any potential association between CXCR5^+^ TILs and tumoral TLS. It is tempting to speculate that CXCR5 facilitates localization within these structures, similar to the role of CXCR5 in positioning CD4^+^ T_fh_ within secondary LNs (48). While only a minority of intratumoral stem-like cells express CXCR5, TCF-1^+^PD-1^lo^CD8^+^ T_ex_ also seem to localize as crude clusters in the TME, implying that there may be additional niche microenvironments within the tumor that support anti-tumor immunity (87). In a histological analysis of prostate, bladder, and kidney cancer biopsies, TCF-1^+^CD8^+^ TILs were predominantly observed within MHC II dense regions, whereas the presumably exhausted TCF-1^−^CD8^+^ TIL appeared to be dispersed (87). Little is known about the role of these MHC II dense niches, which may influence stem-like T cell recruitment and/or dendritic cell (DC) Wnt signaling, thereby maintaining TCF-1 expression and stemness. Stem-like PD-1^lo^CD8^+^ T_ex_ are also preferentially found within tumor-draining secondary LNs over non-draining LNs (89). In contrast, terminally exhausted PD-1^hi^CD8^+^ T_ex_ are predominantly confined to the TME (89). Regardless, if TCF-1^+^PD-1^lo^CD8^+^ T_ex_ infiltrate or expand locally within tumors after systemic delivery of ICB, the intratumoral frequency of these cells can serve as a valuable biomarker to discriminate responders against non-responders (and/or survival within the responder cohort) (85, 90).

## Tissue Distribution and Intratumoral Positioning of Exhausted CD8^+^ T Cells

Tumor PD-L1 expression would logically seem to be a relevant prognostic factor to rationalize the usage of PD-(L)1-based ICB. PD-L1^+^ tumors tend to respond more frequently to anti-PD-(L)1; however, there is only a weak correlation with overall treatment efficacy (33, 91, 92). A significant number of PD-L1^+^ tumors do not respond to ICB, and durable responses are observed in PD-L1^−^ tumors (33, 91). In other analyses, ICB was found to closely align with the raw amount of neoantigens broadly amongst cancers regardless of PD-L1 expression (91, 93–95). Following the completion of The Cancer Genome Atlas (TCGA), a strong correlation was observed between ICB-responsiveness and a T_h_1/IFN-γ inflammatory signature, tumor mutational burden (TMB), and leukocyte infiltration (96). Thus, a combination of a T cell-inflamed gene signature with TMB may currently be the best predictor of ICB-responsiveness (91). PD-L1 expression in the tumor (known to be upregulated by IFN-γ) may reflect tumor inflammation status and thus rather passively indicate an overall immune system status rather than mechanistically predict the response of the tumor to ICB (91).

If inflammation and TMB underlie the response, does ICB act directly in the TME or periphery (97)? Immuno-positron emission tomography (immuno-PET) coupled with blockade of LN egress shows a large portion of effector-like CD8^+^ TIL are derived from the periphery in mice bearing MC38 colorectal tumors systemically treated with anti-PD-1 (98). In the AC29 mesothelioma preclinical model, blockade of LN egress likewise severely compromises the number of CD8^+^ TIL after systemic anti-PD-L1 (89). In the absence of ICB and irrespective of primary tumor PD-L1 expression, enhanced PD-1/PD-L1 contacts between stem-like PD-1^lo^CD8^+^ T_ex_ and migratory PD-L1^+^ DCs entering the paracortex of tumor-draining LNs negatively correlates with survival of mice exposed to AC29 tumors and non-metastatic melanoma patients following resection (89). Localized delivery of anti-PD-L1 to tumor-draining LNs is sufficient to block these interactions and mobilize stem-like CD8^+^ T_ex_ from the lymphatics for proliferation, migration to the TME, and preservation of stemness, leading to an increase in host survival comparable to systemic delivery (89). Further, LN-primed CD8^+^ T_ex_ seem better able to respond to model antigen and proliferate upon *ex vivo* re-stimulation than systemically primed cells (89). These data suggest that LN-primed stem-like CD8^+^ T_ex_ are a critical component of the response to ICB.

Additional studies involving scRNA/TCR-Seq have allowed a more in-depth look at the intratumoral versus peripheral counterparts of immune responses underlying ICB in patients. In a study by Yost et al., scRNA/TCR-Seq analysis of metastatic basal/squamous cell carcinoma patient TIL before and after ICB indicated that clonal replacement dominated the response where upwards of 84% of CD8^+^ T cell clonotypes (having a single TCR specificity) present after treatment were novel (*i.e.*, not present in the tumor before treatment) (72). Intratumoral stem-like TCF-1^+^CD8^+^ T_ex_ did contribute a minor fraction to the population of ICB-activated, tumoricidal clonotypes; however, all cells that attacked tumors again eventually became exhausted (72). Therefore, ICB seems to predominantly mobilize functional CD8^+^ T cells from the periphery into the tumor. A comparison of tumors to normal adjacent tissue (NAT) and peripheral blood *via* scTCR-Seq corroborated these findings across various cancers (99). Patients displaying extratumoral-intratumoral linked clonal expansion across blood/NAT and tumor responded more favorably to ICB (99). However, the action of ICB on stem-like or effector like CD8^+^ T_ex_ inside the tumor cannot be dismissed. Despite the lack of a significant correlation between intratumoral PD-L1 expression and survival, PD-1/PD-L1 interactions in the tumor as measured by immune-Förster resonance energy transfer (iFRET) is more predictive of survival in metastatic melanoma and NSCLC patients receiving ICB, in line with findings in draining LNs (89, 100).

Emergent data suggest that the CCR5 and CXCR3 chemokine receptor pathways are needed for anti-PD-(L)1-mediated CD8^+^ T_ex_ tumor recruitment and/or intratumoral positioning (101–104). Heightened dual expression of the ligands for CCR5 and CXCR3 (CCL5 and CXCL9, respectively) positively correlates with the amount of tumor *CD8a* transcripts and patient survival in cancers of the ovary, breast, lung, colon, as well as melanoma (103). CCL5 from tumor cells or tumor-associated myeloid cells appears to license CXCL9 production almost exclusively from inflammatory CD68^+^ macrophages and CD11c^+^ DCs within the TME (98, 103, 105, 106). Genetic deletion or antibody-mediated blockade of either CCL5 and CXCL9 significantly compromises CD8^+^ T cell recruitment to the TME; however, only CXCL9 correlates with ICB efficacy in multiple preclinical models (102–105). Revisiting CD8^+^ T_ex_ CCR5 and CXCR3 progenitor/progeny expression patterns in the Clone 13 and preclinical tumor models may clarify this. In both settings, CXCR3 is predominantly expressed on stem-like PD-1^lo^CD8^+^ T_ex_ (**Figures 4B, C**), whereas CCR5 is oppositely elevated on terminally exhausted PD-1^hi^CD8^+^ T_ex_ (**Figure 4E**) (46, 69, 102). These axes may be necessary for the positioning and stability of stem-like PD-1^lo^CD8^+^ T_ex_ in the previously mentioned intratumoral MHC II dense clusters by undescribed mechanisms or serve as markers for recent PD-1^lo^ versus PD-1^hi^CD8^+^ T_ex_ CXCR3-mediated trafficking (87, 107, 108). With LN egress blocked, anti-PD-1 was shown to directly increase the expansion of intratumoral wild type but not *Cxcr3* ^−/−^ CD8^+^ T cells, which may be related to localization of these cells within the TME or intrinsic effects (102). CXCR3 itself is known to support T-bet expression and favor SLEC differentiation during acute infection and may play a direct role in dictating stem-like to transitory CD8^+^ T_ex_ differentiation (109, 110). Therapies centered on CXCR3 agonism may augment CD8^+^ T cell trafficking, positioning, and priming/expansion depending on the exact intersection with the T_ex_ lineage.

## Clinical Perspectives

Despite the undisputed success of ICB in the clinic, it may one day be replaced or combined with other immunotherapies due to its inherent failure in preventing exhaustion. Our increasingly granular understanding of CD8^+^ T_ex_ and the underlying regulatory mechanisms may present novel therapeutic avenues that include alternative ways to stimulate and stabilize stem/effector-like states along the exhaustion continuum or enhance memory cell lineage commitment. Simple amplification of CD8^+^ T cell responses by modulating trafficking and tumor positioning may stabilize stem-like and effector-like transitory CD8^+^ T_ex_. Durable responses may also be possible if effector-like CD8^+^ T cells can instead be directly coerced to persist in the transitory cytolytic state, for instance, by pharmacologically antagonizing TOX or related mediators of exhaustion. In addition, as stem-like PD-1^lo^CD8^+^ T_ex_ exhibit heightened expression of several members of the immunoglobulin and tumor-necrosis factor receptor (TNFR) superfamily, including *Tnfrsf4* (OX40) and *Tnfrsf9* (4-1BB), combining ICB with TNFR superfamily member agonism may further support long-lived CD8^+^ T cell reinvigoration by preferentially targeting the stem-like subset (47). Inhibiting other known or unknown transcriptional components or downstream effector pathways of the exhaustion program may offer other therapeutic avenues.

If maintaining stabilized anti-tumoral CD8^+^ T cells is impossible, maximal amplification of the response *via* focused neoantigen vaccination or repetitive infusions of adoptive cellular therapies (ACT) may be warranted. Today, it is possible to administer autologous CD8^+^ T cells genetically engineered to express neoantigen-specific TCRs or chimeric antigen receptors (CARs) (111, 112). This may allow for an unlimited source of artificially generated anti-tumoral CD8^+^ T cells, thus bypassing the challenge that exhaustion may be unavoidable. ACT may also be designed to be exhaustion-resistant or to maintain stemness through gene-editing technologies (112). Alternatively, neoantigen vaccination might be a more promising strategy, either as part of a patient-shared or fully personalized therapeutic approach (113, 114). Neoantigen vaccines carrying both CD4^+^ and CD8^+^ T cell epitopes as long peptides, RNA/DNA vectors, or within viral constructs may better support robust, helper-primed CD8^+^ T cell responses able to resist exhaustion upon repeated antigen encounter (115–121). Neoantigen vaccination can also strategically address tumor immunoediting. Even if persistent antigens are effectively cleared, some residual tumor cells can unavoidably become resistant to first-line ICB and/or neoantigen vaccination by altering MHC I-displayed tumor antigens *via* deletion or mutation (122). Neoantigen vaccination can solve this by applying booster regimens modified in real-time against resistant tumor cell clonal outgrowth.

## Conclusion

Understanding how tumors shape CD8^+^ T cell exhaustion is needed to effectively program the immune system to destroy cancer—the professed ‘emperor of all maladies’ (123). An exciting parallel journey between chronic viral infection and cancer has thus been embarked upon to bypass exhaustion and identify the causative molecular cues, new cell types/lineages permissive to ICB, and innovative paths for immunotherapeutic strategies. It is currently clear that reversing exhaustion in PD-1^hi^CD8^+^ T_ex_ is unlikely. Selective mobilization of stem-like CD8^+^ T_ex_ is instead called for and lies at the crux of generating functional and stable anti-tumor immune responses. Besides re-shaping the CD8^+^ T_ex_ developmental continuum, scientists are dually challenged with directing specificity of the responding population as ICB also relies on the endogenous immune system for spontaneous recognition of select neoantigens from an initially broad TCR repertoire (90, 124, 125). Can stem-like and effector-like CD8^+^ T_ex_ fates be stabilized to act as a continuous source to deliver an unending supply of tumoricidal CD8^+^ T cells? Can exhaustion itself be prevented in response to ICB? Can chemokine receptor pathways be exploited to control TME positioning and differentiation status of intratumoral CD8^+^ T_ex_? Or should immunologists accept the demise of CD8^+^ T_ex_ and deploy patient-tailored neoantigen and ACT strategies? The answers to these outstanding questions undoubtedly lay forth the path of future clinical trials.

## Author Contributions

All authors conceived, discussed content, and contributed to researching data for the article. JD produced the primary drafts of the manuscript and designed the figures. NB-B, GT, and SS-A. provided writing and editorial contributions. All authors contributed to the article and approved the submitted version.

## Funding

The authors declare that this study received funding from Pfizer. The funder was not involved in the study design, collection, analysis, interpretation of data, the writing of this article, or the decision to submit it for publication.

## Conflict of Interest

All authors are employees of Pfizer, Inc. and hold stock/stock options in the company.

## References

[1] Blank CU Haining WN Held W Hogan PG Kallies A Lugli E. Defining ‘T Cell Exhaustion’. Nat Rev Immunol (2019) 14:768. 10.1038/s41577-019-0221-9 PMC7286441 31570879

[2] Virgin HW Wherry EJ Ahmed R. Redefining Chronic Viral Infection. Cell (2009) 138:30–50. 10.1016/j.cell.2009.06.036 19596234

[3] Thommen DS Schumacher TN. T Cell Dysfunction in Cancer. Cancer Cell (2018) 33:547–62. 10.1016/j.ccell.2018.03.012 PMC7116508 29634943

[4] McLane LM Abdel-Hakeem MS Wherry EJ. Cd8 T Cell Exhaustion During Chronic Viral Infection and Cancer. Annu Rev Immunol (2019) 37:457–95. 10.1146/annurev-immunol-041015-055318 30676822

[5] Klein MR van der Burg SH Pontesilli O Miedema F. Cytotoxic T Lymphocytes in HIV-1 Infection: A Killing Paradox? Immunol Today (1998) 19:317–24. 10.1016/s0167-5699(98)01288-2 9666605

[6] Obar JJ Lefrançois L. Memory CD8+ T Cell Differentiation. Ann NY Acad Sci (2010) 1183:251–66. 10.1111/j.1749-6632.2009.05126.x PMC2836783 20146720

[7] Seder RA Ahmed R. Similarities and Differences in CD4+ and CD8+ Effector and Memory T Cell Generation. Nat Immunol (2003) 4:835–42. 10.1038/ni969 12942084

[8] Joshi NS Cui W Chandele A Lee HK Urso DR Hagman J. Inflammation Directs Memory Precursor and Short-Lived Effector Cd8+ T Cell Fates Via the Graded Expression of T-Bet Transcription Factor. Immunity (2007) 27:281–95. 10.1016/j.immuni.2007.07.010 PMC2034442 17723218

[9] Moskophidis D Lechner F Pircher H Zinkernagel RM. Virus Persistence in Acutely Infected Immunocompetent Mice by Exhaustion of Antiviral Cytotoxic Effector T Cells. Nature (1993) 362:758–61. 10.1038/362758a0 8469287

[10] Wherry EJ Blattman JN Murali-Krishna K van der Most R Ahmed R. Viral Persistence Alters CD8 T-Cell Immunodominance and Tissue Distribution and Results in Distinct Stages of Functional Impairment. J Virol (2003) 77:4911–27. 10.1128/JVI.77.8.4911-4927.2003 PMC152117 12663797

[11] Zajac AJ Blattman JN Murali-Krishna K Sourdive DJ Suresh M Altman JD. Viral Immune Evasion Due to Persistence of Activated T Cells Without Effector Function. J Exp Med (1998) 188:2205–13. 10.1084/jem.188.12.2205 PMC2212420 9858507

[12] Gallimore A Dumrese T Hengartner H Zinkernagel RM Rammensee HG. Protective Immunity Does Not Correlate With the Hierarchy of Virus-Specific Cytotoxic T Cell Responses to Naturally Processed Peptides. J Exp Med (1998) 187:1647–57. 10.1084/jem.187.10.1647-b PMC2212291 9584143

[13] Klenerman P Hill A. T Cells and Viral Persistence: Lessons From Diverse Infections. Nat Immunol (2005) 6:873–9. 10.1038/ni1241 16116467

[14] Hellström I Hellström KE Pierce GE Yang JP. Cellular and Humoral Immunity to Different Types of Human Neoplasms. Nature (1968) 220:1352–4. 10.1038/2201352a0 4302696

[15] Tran E Robbins PF Lu Y-C Prickett TD Gartner JJ Jia L. T-Cell Transfer Therapy Targeting Mutant KRAS in Cancer. N Engl J Med (2016) 375:2255–62. 10.1056/NEJMoa1609279 PMC5178827 27959684

[16] Rosenberg SA. Progress in the Development of Immunotherapy for the Treatment of Patients With Cancer. J Intern Med (2001) 250:462–75. 10.1046/j.1365-2796.2001.00911.x PMC2413437 11902815

[17] Mognol GP Spreafico R Wong V Scott-Browne JP Togher S Hoffmann A. Exhaustion-Associated Regulatory Regions in CD8+ Tumor-Infiltrating T Cells. Proc Natl Acad Sci USA (2017) 114:E2776–85. 10.1073/pnas.1620498114 PMC5380094 28283662

[18] Schietinger A Philip M Krisnawan VE Chiu EY Delrow JJ Basom RS. Tumor-Specific T Cell Dysfunction Is a Dynamic Antigen-Driven Differentiation Program Initiated Early During Tumorigenesis. Immunity (2016) 45:389–401. 10.1016/j.immuni.2016.07.011 27521269 PMC5119632

[19] Ahrends T Spanjaard A Pilzecker B Bąbała N Bovens A Xiao Y. CD4+ T Cell Help Confers a Cytotoxic T Cell Effector Program Including Coinhibitory Receptor Downregulation and Increased Tissue Invasiveness. Immunity (2017) 47:848–61.e5. 10.1016/j.immuni.2017.10.009 29126798

[20] Martinez GJ Pereira RM Äijö T Kim EY Marangoni F Pipkin ME. The Transcription Factor NFAT Promotes Exhaustion of Activated CD8⁺ T Cells. Immunity (2015) 42:265–78. 10.1016/j.immuni.2015.01.006 PMC4346317 25680272

[21] Pauken KE Sammons MA Odorizzi PM Manne S Godec J Khan O. Epigenetic Stability of Exhausted T Cells Limits Durability of Reinvigoration by PD-1 Blockade. Science (2016) 354:1160–5. 10.1126/science.aaf2807 PMC5484795 27789795

[22] Pipkin ME Sacks JA Cruz-Guilloty F Lichtenheld MG Bevan MJ Rao A. Interleukin-2 and Inflammation Induce Distinct Transcriptional Programs That Promote the Differentiation of Effector Cytolytic T Cells. Immunity (2010) 32:79–90. 10.1016/j.immuni.2009.11.012 20096607 PMC2906224

[23] Ashouri JF Weiss A. Endogenous Nur77 Is a Specific Indicator of Antigen Receptor Signaling in Human T and B Cells. J Immunol (2017) 198:657–68. 10.4049/jimmunol.1601301 PMC5224971 27940659

[24] Khan O Giles JR McDonald S Manne S Ngiow SF Patel KP. TOX Transcriptionally and Epigenetically Programs CD8+ T Cell Exhaustion. Nature (2019) 571:211–8. 10.1038/s41586-019-1325-x PMC6713202 31207603

[25] Man K Gabriel SS Liao Y Gloury R Preston S Henstridge DC. Transcription Factor Irf4 Promotes CD8+ T Cell Exhaustion and Limits the Development of Memory-Like T Cells During Chronic Infection. Immunity (2017) 47:1129–41.e5. 10.1016/j.immuni.2017.11.021 29246443

[26] Chen J López-Moyado IF Seo H Lio C-WJ Hempleman LJ Sekiya T. NR4A Transcription Factors Limit CAR T Cell Function in Solid Tumours. Nature (2019) 567:530–4. 10.1038/s41586-019-0985-x PMC6546093 30814732

[27] Zhao M Kiernan CH Stairiker CJ Hope JL Leon LG van Meurs M. Rapid In Vitro Generation of Bona Fide Exhausted CD8+ T Cells Is Accompanied by Tcf7 promotor Methylation. PloS Pathog (2020) 16:e1008555. 10.1371/journal.ppat.1008555 32579593 PMC7340326

[28] Thommen DS Koelzer VH Herzig P Roller A Trefny M Dimeloe S. A Transcriptionally and Functionally Distinct PD-1+ CD8+ T Cell Pool With Predictive Potential in Non-Small-Cell Lung Cancer Treated With PD-1 Blockade. Nat Med (2018) 24:994–1004. 10.1038/s41591-018-0057-z 29892065 PMC6110381

[29] Keir ME Freeman GJ Sharpe AH. PD-1 Regulates Self-Reactive CD8+ T Cell Responses to Antigen in Lymph Nodes and Tissues. J Immunol (2007) 179:5064–70. 10.4049/jimmunol.179.8.5064 17911591

[30] Sharpe AH Wherry EJ Ahmed R Freeman GJ. The Function of Programmed Cell Death 1 and Its Ligands in Regulating Autoimmunity and Infection. Nat Immunol (2007) 8:239–45. 10.1038/ni1443 17304234

[31] Barber DL Wherry EJ Masopust D Zhu B Allison JP Sharpe AH. Restoring Function in Exhausted CD8 T Cells During Chronic Viral Infection. Nature (2006) 439:682–7. 10.1038/nature04444 16382236

[32] de Miguel M Calvo E. Clinical Challenges of Immune Checkpoint Inhibitors. Cancer Cell (2020) 38:326–33. 10.1016/j.ccell.2020.07.004 32750319

[33] Postow MA Callahan MK Wolchok JD. Immune Checkpoint Blockade in Cancer Therapy. J Clin Oncol (2015) 33:1974–82. 10.1200/JCO.2014.59.4358 PMC4980573 25605845

[34] Blackburn SD Shin H Freeman GJ Wherry EJ. Selective Expansion of a Subset of Exhausted CD8 T Cells by alphaPD-L1 Blockade. Proc Natl Acad Sci USA (2008) 105:15016–21. 10.1073/pnas.0801497105 PMC2567485 18809920

[35] Blackburn SD Shin H Haining WN Zou T Workman CJ Polley A. Coregulation of CD8+ T Cell Exhaustion by Multiple Inhibitory Receptors During Chronic Viral Infection. Nat Immunol (2009) 10:29–37. 10.1038/ni.1679 19043418 PMC2605166

[36] Jin H-T Anderson AC Tan WG West EE Ha S-J Araki K. Cooperation of Tim-3 and PD-1 in CD8 T-Cell Exhaustion During Chronic Viral Infection. Proc Natl Acad Sci USA (2010) 107:14733–8. 10.1073/pnas.1009731107 PMC2930455 20679213

[37] McMahan RH Golden-Mason L Nishimura MI McMahon BJ Kemper M Allen TM. Tim-3 Expression on PD-1+ HCV-Specific Human CTLs Is Associated With Viral Persistence, and its Blockade Restores Hepatocyte-Directed In Vitro Cytotoxicity. J Clin Invest (2010) 120:4546–57. 10.1172/JCI43127DS1 PMC2994339 21084749

[38] Nakamoto N Cho H Shaked A Olthoff K Valiga ME Kaminski M. Synergistic Reversal of Intrahepatic HCV-Specific Cd8 T Cell Exhaustion by Combined PD-1/CTLA-4 Blockade. PloS Pathog (2009) 5:e1000313. 10.1371/journal.ppat.1000313 19247441 PMC2642724

[39] Chung HK McDonald B Kaech SM. The Architectural Design of CD8+ T Cell Responses in Acute and Chronic Infection: Parallel Structures With Divergent Fates. J Exp Med (2021) 218:e20201730. 10.1084/jem.20201730 33755719 PMC7992501

[40] McLane LM Ngiow SF Chen Z Attanasio J Manne S Ruthel G. Role of Nuclear Localization in the Regulation and Function of T-Bet and Eomes in Exhausted CD8 T Cells. CellReports (2021) 35:109120. 10.1016/j.celrep.2021.109120 PMC8195461 33979613

[41] Paley MA Kroy DC Odorizzi PM Johnnidis JB Dolfi DV Barnett BE. Progenitor and Terminal Subsets of CD8+ T Cells Cooperate to Contain Chronic Viral Infection. Science (2012) 338:1220–5. 10.1126/science.1229620 PMC3653769 23197535

[42] Chang JT Palanivel VR Kinjyo I Schambach F Intlekofer AM Banerjee A. Asymmetric T Lymphocyte Division in the Initiation of Adaptive Immune Responses. Science (2007) 315:1687–91. 10.1126/science.1139393 17332376

[43] Chang JT Ciocca ML Kinjyo I Palanivel VR McClurkin CE DeJong CS. Asymmetric Proteasome Segregation as a Mechanism for Unequal Partitioning of the Transcription Factor T-Bet During T Lymphocyte Division. Immunity (2011) 34:492–504. 10.1016/j.immuni.2011.03.017 21497118 PMC3088519

[44] Obar JJ Lefrancois L. Early Events Governing Memory CD8+ T-Cell Differentiation. Int Immunol (2010) 22:619–25. 10.1093/intimm/dxq053 PMC2908475 20504887

[45] Rao RR Li Q Odunsi K Shrikant PA. The Mtor Kinase Determines Effector Versus Memory CD8+ T Cell Fate by Regulating the Expression of Transcription Factors T-bet and Eomesodermin. Immunity (2010) 32:67–78. 10.1016/j.immuni.2009.10.010 20060330 PMC5836496

[46] Beltra J-C Manne S Abdel-Hakeem MS Kurachi M Giles JR Chen Z. Developmental Relationships of Four Exhausted CD8+ T Cell Subsets Reveals Underlying Transcriptional and Epigenetic Landscape Control Mechanisms. Immunity (2020) 52:825–41.e8. 10.1016/j.immuni.2020.04.014 32396847 PMC8360766

[47] Im SJ Hashimoto M Gerner MY Lee J Kissick HT Burger MC. Defining CD8+ T Cells That Provide the Proliferative Burst After PD-1 Therapy. Nature (2016) 537:417–21. 10.1038/nature19330 PMC5297183 27501248

[48] Crotty S. Follicular Helper CD4 T Cells (TFH). Annu Rev Immunol (2011) 29:621–63. 10.1146/annurev-immunol-031210-101400 21314428

[49] Escobar G Mangani D Anderson AC. T Cell Factor 1: A Master Regulator of the T Cell Response in Disease. Sci Immunol (2020) 5:eabb9726. 10.1126/sciimmunol.abb9726 33158974 PMC8221367

[50] Pais Ferreira D Silva JG Wyss T Fuertes Marraco SA Scarpellino L Charmoy M. Central Memory CD8+ T Cells Derive From Stem-Like Tcf7hi Effector Cells in the Absence of Cytotoxic Differentiation. Immunity (2020) 53:985–1000. 10.1016/j.immuni.2020.09.005 33128876

[51] Wu T Ji Y Moseman EA Xu HC Manglani M Kirby M. The TCF1-Bcl6 Axis Counteracts Type I Interferon to Repress Exhaustion and Maintain T Cell Stemness. Sci Immunol (2016) 1:eaai8593–eaai8593. 10.1126/sciimmunol.aai8593 28018990 PMC5179228

[52] Hwang S Cobb DA Bhadra R Youngblood B Khan IA. Blimp-1-mediated Cd4 T Cell Exhaustion Causes CD8 T Cell Dysfunction During Chronic Toxoplasmosis. J Exp Med (2016) 213:1799–818. 10.1084/jem.20151995 PMC4995081 27481131

[53] Tirosh I Izar B Prakadan SM Wadsworth MH Treacy D Trombetta JJ. Dissecting the Multicellular Ecosystem of Metastatic Melanoma by Single-Cell RNA-Seq. Science (2016) 352:189–96. 10.1126/science.aad0501 PMC4944528 27124452

[54] Wherry EJ Ha S-J Kaech SM Haining WN Sarkar S Kalia V. Molecular Signature of CD8+ T Cell Exhaustion During Chronic Viral Infection. Immunity (2007) 27:670–84. 10.1016/j.immuni.2007.09.006 17950003

[55] Shan Q Hu S Chen X Danahy DB Badovinac VP Zang C. Ectopic Tcf1 Expression Instills a Stem-Like Program in Exhausted CD8+ T Cells to Enhance Viral and Tumor Immunity. Cell Mol Immunol (2020) 58:89. 10.1038/s41423-020-0436-5 PMC8093427 32341523

[56] Alfei F Kanev K Hofmann M Wu M Ghoneim HE Roelli P. TOX Reinforces the Phenotype and Longevity of Exhausted T Cells in Chronic Viral Infection. Nature (2019) 571:265–9. 10.1038/s41586-019-1326-9 31207605

[57] Utzschneider DT Gabriel SS Chisanga D Gloury R Gubser PM Vasanthakumar A. Early Precursor T Cells Establish and Propagate T Cell Exhaustion in Chronic Infection. Nat Immunol (2020) 21:1256–66. 10.1038/s41590-020-0760-z 32839610

[58] Utzschneider DT Legat A Fuertes Marraco SA Carrié L Luescher I Speiser DE. T Cells Maintain an Exhausted Phenotype After Antigen Withdrawal and Population Reexpansion. Nat Immunol (2013) 14:603–10. 10.1038/ni.2606 23644506

[59] Yao C Lou G Sun H-W Zhu Z Sun Y Chen Z. BACH2 Enforces the Transcriptional and Epigenetic Programs of Stem-Like CD8+ T Cells. Nat Immunol (2021) 22:370–80. 10.1038/s41590-021-00868-7 PMC7906956 33574619

[60] Lugli E Galletti G Boi SK Youngblood BA. Stem, Effector, and Hybrid States of Memory CD8+ T Cells. Trends Immunol (2020) 41:17–28. 10.1016/j.it.2019.11.004 31810790 PMC6934921

[61] Pritykin Y van der Veeken J Pine AR Zhong Y Sahin M Mazutis L. A Unified Atlas of CD8 T Cell Dysfunctional States in Cancer and Infection. Mol Cell (2021) 81:2477–93. 10.1016/j.molcel.2021.03.045 PMC8454502 33891860

[62] Galletti G De Simone G Mazza EMC Puccio S Mezzanotte C Bi TM. Two Subsets of Stem-Like CD8+ Memory T Cell Progenitors With Distinct Fate Commitments in Humans. Nat Immunol (2020) 2:251. 10.1038/s41590-020-0791-5 PMC7610790 33046887

[63] Sen DR Kaminski J Barnitz RA Kurachi M Gerdemann U Yates KB. The Epigenetic Landscape of T Cell Exhaustion. Science (2016) 354:1165–9. 10.1126/science.aae0491 PMC5497589 27789799

[64] Zeng Z Wei F Ren X. Exhausted T Cells and Epigenetic Status. Cancer Biol Med (2020) 17:923–36. 10.20892/j.issn.2095-3941.2020.0338 PMC7721092 33299644

[65] Scott AC Dündar F Zumbo P Chandran SS Klebanoff CA Shakiba M. TOX Is a Critical Regulator of Tumour-Specific T Cell Differentiation. Nature (2019) 571:270–4. 10.1038/s41586-019-1324-y PMC7698992 31207604

[66] Seo H Chen J González-Avalos E Samaniego-Castruita D Das A Wang YH. TOX and TOX2 Transcription Factors Cooperate With NR4A Transcription Factors to Impose CD8+ T Cell Exhaustion. Proc Natl Acad Sci USA (2019) 116:12410–5. 10.1073/pnas.1905675116 PMC6589758 31152140

[67] Yao C Sun H-W Lacey NE Ji Y Moseman EA Shih H-Y. Single-Cell RNA-Seq Reveals TOX as a Key Regulator of CD8+ T Cell Persistence in Chronic Infection. Nat Immunol (2019) 20:890–901. 10.1038/s41590-019-0403-4 31209400 PMC6588409

[68] Chen Z Ji Z Ngiow SF Manne S Cai Z Huang AC. Tcf-1-Centered Transcriptional Network Drives an Effector Versus Exhausted CD8 T Cell-Fate Decision. Immunity (2019) 51:1–16. 10.1016/j.immuni.2019.09.013 31606264 PMC6943829

[69] Hudson WH Gensheimer J Hashimoto M Wieland A Valanparambil RM Li P. Proliferating Transitory T Cells With an Effector-like Transcriptional Signature Emerge From PD-1+ Stem-Like CD8+ T Cells During Chronic Infection. Immunity (2019) 51:1043–58. 10.1016/j.immuni.2019.11.002 PMC6920571 31810882

[70] Zhou J Wang W Liang Z Ni B He W Wang D. Clinical Significance of CD38 and CD101 Expression in PD-1+CD8+ T Cells in Patients With Epithelial Ovarian Cancer. Oncol Lett (2020) 20:724–32. 10.3892/ol.2020.11580 PMC7285834 32565998

[71] Kurtulus S Madi A Escobar G Klapholz M Nyman J Christian E. Checkpoint Blockade Immunotherapy Induces Dynamic Changes in PD-1-CD8+ Tumor-Infiltrating T Cells. Immunity (2019) 50:181–94. 10.1016/j.immuni.2018.11.014 PMC6336113 30635236

[72] Yost KE Satpathy AT Wells DK Qi Y Wang C Kageyama R. Clonal Replacement of Tumor-Specific T Cells Following PD-1 Blockade. Nat Med (2019) 348:56. 10.1038/s41591-019-0522-3 PMC6689255 31359002

[73] Xu T Keller A Martinez GJ. NFAT1 and NFAT2 Differentially Regulate Ctl Differentiation Upon Acute Viral Infection. Front Immunol (2019) 10:184. 10.3389/fimmu.2019.00184 30828328 PMC6384247

[74] Ahrends T Bąbała N Xiao Y Yagita H van Eenennaam H Borst J. Cd27 Agonism Plus PD-1 Blockade Recapitulates CD4+ T-Cell Help in Therapeutic Anticancer Vaccination. Cancer Res (2016) 76:2921–31. 10.1158/0008-5472.CAN-15-3130 27020860

[75] Borst J Ahrends T Bąbała N Melief CJM Kastenmüller W. CD4+ T Cell Help in Cancer Immunology and Immunotherapy. Nat Rev Immunol (2018) 18:635–47. 10.1038/s41577-018-0044-0 30057419

[76] Croft M. Co-Stimulatory Members of the TNFR Family: Keys to Effective T-Cell Immunity? Nat Rev Immunol (2003) 3:609–20. 10.1038/nri1148 12974476

[77] Kong K-F Yokosuka T Canonigo-Balancio AJ Isakov N Saito T Altman A. A Motif in the V3 Domain of the Kinase PKC-θ Determines Its Localization in the Immunological Synapse and Functions in T Cells Via Association With CD28. Nat Publishing Group (2011) 12:1105–12. 10.1038/ni.2120 PMC3197934 21964608

[78] Kong K-F Altman A. In and Out of the Bull’s Eye: Protein Kinase Cs in the Immunological Synapse. Trends Immunol (2013) 34:234–42. 10.1016/j.it.2013.01.002 PMC3647024 23428395

[79] Macian F García-Rodríguez C Rao A. Gene Expression Elicited by NFAT in the Presence or Absence of Cooperative Recruitment of Fos and Jun. EMBO J (2000) 19:4783–95. 10.1093/emboj/19.17.4783 PMC302068 10970869

[80] Macián F García-Cózar F Im S-H Horton HF Byrne MC Rao A. Transcriptional Mechanisms Underlying Lymphocyte Tolerance. Cell (2002) 109:719–31. 10.1016/s0092-8674(02)00767-5 12086671

[81] Franco F Jaccard A Romero P Yu Y-R Ho P-C. Metabolic and Epigenetic Regulation of T-Cell Exhaustion. Nat Metab (2020) 2:1001–12. 10.1038/s42255-020-00280-9 32958939

[82] Vardhana SA Hwee MA Berisa M Wells DK Yost KE King B. Impaired Mitochondrial Oxidative Phosphorylation Limits the Self-Renewal of T Cells Exposed to Persistent Antigen. Nat Immunol (2020) 21:1022–33. 10.1038/s41590-020-0725-2 PMC7442749 32661364

[83] Yu Y-R Imrichova H Wang H Chao T Xiao Z Gao M. Disturbed Mitochondrial Dynamics in CD8+ Tils Reinforce T Cell Exhaustion. Nat Immunol (2020) 16:425. 10.1038/s41590-020-0793-3 33020660

[84] Sena LA Li S Jairaman A Prakriya M Ezponda T Hildeman DA. Mitochondria Are Required for Antigen-Specific T Cell Activation Through Reactive Oxygen Species Signaling. Immunity (2013) 38:225–36. 10.1016/j.immuni.2012.10.020 PMC3582741 23415911

[85] Sade-Feldman M Yizhak K Bjorgaard SL Ray JP de Boer CG Jenkins RW. Defining T Cell States Associated With Response to Checkpoint Immunotherapy in Melanoma. Cell (2018) 175:998–1013. 10.1016/j.cell.2018.10.038 30388456 PMC6641984

[86] Miller BC Sen DR Abosy Al R Bi K Virkud YV LaFleur MW. Subsets of Exhausted CD8+ T Cells Differentially Mediate Tumor Control and Respond to Checkpoint Blockade. Nat Immunol (2019) 20:326–36. 10.1038/s41590-019-0312-6 PMC6673650 30778252

[87] Jansen CS Prokhnevska N Master VA Sanda MG Carlisle JW Bilen MA. An Intra-Tumoral Niche Maintains and Differentiates Stem-Like CD8 T Cells. Nature (2019) 576:465–70. 10.1038/s41586-019-1836-5 PMC7108171 31827286

[88] Brummelman J Mazza EMC Alvisi G Colombo FS Grilli A Mikulak J. High-Dimensional Single Cell Analysis Identifies Stem-Like Cytotoxic CD8+ T Cells Infiltrating Human Tumors. J Exp Med (2018) 215:2520–35. 10.1084/jem.20180684 PMC6170179 30154266

[89] Dammeijer F van Gulijk M Mulder EE Lukkes M Klaase L van den Bosch T. The PD-1/PD-L1-Checkpoint Restrains T Cell Immunity in Tumor-Draining Lymph Nodes. Cancer Cell (2020) 38:685–700. 10.1016/j.ccell.2020.09.001 33007259

[90] Ott PA Hu-Lieskovan S Chmielowski B Govindan R Naing A Bhardwaj N. A Phase Ib Trial of Personalized Neoantigen Therapy Plus Anti-PD-1 in Patients With Advanced Melanoma, Non-small Cell Lung Cancer, or Bladder Cancer. Cell (2020) 183:347–62.e24. 10.1016/j.cell.2020.08.053 33064988

[91] Cristescu R Mogg R Ayers M Albright A Murphy E Yearley J. Pan-Tumor Genomic Biomarkers for PD-1 Checkpoint Blockade-Based Immunotherapy. Science (2018) 362:eaar3593. 10.1126/science.aar3593 30309915 PMC6718162

[92] Davis AA Patel VG. The Role of PD-L1 Expression as a Predictive Biomarker: An Analysis of All US Food and Drug Administration (FDA) Approvals of Immune Checkpoint Inhibitors. J Immunother Cancer (2019) 7:278. 10.1186/s40425-019-0768-9 31655605 PMC6815032

[93] Rizvi NA Hellmann MD Snyder A Kvistborg P Makarov V Havel JJ. Mutational Landscape Determines Sensitivity to PD-1 Blockade in non-Small Cell Lung Cancer. Science (2015) 348:124–8. 10.1126/science.aaa1348 PMC4993154 25765070

[94] Schumacher TN Schreiber RD. Neoantigens in Cancer Immunotherapy. Science (2015) 348:69–74. 10.1126/science.aaa4971 25838375

[95] Snyder A Makarov V Merghoub T Yuan J Zaretsky JM Desrichard A. Genetic Basis for Clinical Response to CTLA-4 Blockade in Melanoma. N Engl J Med (2014) 371:2189–99. 10.1056/NEJMoa1406498 PMC4315319 25409260

[96] Thorsson V Gibbs DL Brown SD Wolf D Bortone DS Ou Yang T-H. The Immune Landscape of Cancer. Immunity (2018) 48:812–30.e14. 10.1016/j.immuni.2018.03.023 29628290 PMC5982584

[97] Yost KE Chang HY Satpathy AT. Recruiting T Cells in Cancer Immunotherapy. Science (2021) 372:130–1. 10.1126/science.abd1329 33833111

[98] Rashidian M LaFleur MW Verschoor VL Dongre A Zhang Y Nguyen TH. Immuno-PET Identifies the Myeloid Compartment as a Key Contributor to the Outcome of the Antitumor Response Under PD-1 Blockade. Proc Natl Acad Sci USA (2019) 116:16971–80. 10.1073/pnas.1905005116 PMC6708368 31375632

[99] Wu TD Madireddi S de Almeida PE Banchereau R Chen Y-JJ Chitre AS. Peripheral T Cell Expansion Predicts Tumour Infiltration and Clinical Response. Nature (2020) 579:274–8. 10.1038/s41586-020-2056-8 32103181

[100] Sánchez-Magraner L Miles J Baker CL Applebee CJ Lee D-J Elsheikh S. High PD-1/PD-L1 Checkpoint Interaction Infers Tumor Selection and Therapeutic Sensitivity to Anti-PD-1/PD-L1 Treatment. Cancer Res (2020) 80:4244–57. 10.1158/0008-5472.CAN-20-1117 32855204

[101] Chen IX Newcomer K Pauken KE Juneja VR Naxerova K Wu MW. A Bilateral Tumor Model Identifies Transcriptional Programs Associated With Patient Response to Immune Checkpoint Blockade. Proc Natl Acad Sci USA (2020) 2:202002806. 10.1073/pnas.2002806117 PMC7519254 32907939

[102] Chow MT Ozga AJ Servis RL Frederick DT Lo JA Fisher DE. Intratumoral Activity of the CXCR3 Chemokine System Is Required for the Efficacy of Anti-PD-1 Therapy. Immunity (2019) 50:1498–512.e5. 10.1016/j.immuni.2019.04.010 31097342 PMC6527362

[103] Dangaj D Bruand M Grimm AJ Ronet C Barras D Duttagupta PA. Cooperation Between Constitutive and Inducible Chemokines Enables T Cell Engraftment and Immune Attack in Solid Tumors. Cancer Cell (2019) 35:885–900.e10. 10.1016/j.ccell.2019.05.004 31185212 PMC6961655

[104] House IG Savas P Lai J Chen AXY Oliver AJ Teo ZL. Macrophage-Derived CXCL9 and CXCL10 Are Required for Antitumor Immune Responses Following Immune Checkpoint Blockade. Clin Cancer Res (2020) 26:487–504. 10.1158/1078-0432.CCR-19-1868 31636098

[105] Qu Y Wen J Thomas G Yang W Prior W He W. Baseline Frequency of Inflammatory Cxcl9-Expressing Tumor-Associated Macrophages Predicts Response to Avelumab Treatment. CellReports (2020) 32:107873. 10.1016/j.celrep.2020.107873 32640238

[106] Zilionis R Engblom C Pfirschke C Savova V Zemmour D Saatcioglu HD. Single-Cell Transcriptomics of Human and Mouse Lung Cancers Reveals Conserved Myeloid Populations Across Individuals and Species. Immunity (2019) 50:1317–34.e10. 10.1016/j.immuni.2019.03.009 30979687 PMC6620049

[107] Abboud G Desai P Dastmalchi F Stanfield J Tahiliani V Hutchinson TE. Tissue-Specific Programming of Memory CD8 T Cell Subsets Impacts Protection Against Lethal Respiratory Virus Infection. J Exp Med (2016) 213:2897–911. 10.1084/jem.20160167 PMC5154936 27879287

[108] Desai P Tahiliani V Stanfield J Abboud G Salek-Ardakani S. Inflammatory Monocytes Contribute to the Persistence of CXCR3hi CX3CR1lo Circulating and Lung-Resident Memory CD8+ T Cells Following Respiratory Virus Infection. Immunol Cell Biol (2018) 96:370–8. 10.1111/imcb.12006 PMC5916332 29363162

[109] Kurachi M Kurachi J Suenaga F Tsukui T Abe J Ueha S. Chemokine Receptor CXCR3 Facilitates CD8(+) T Cell Differentiation Into Short-Lived Effector Cells Leading to Memory Degeneration. J Exp Med (2011) 208:1605–20. 10.1084/jem.20102101 PMC3149224 21788406

[110] Tokunaga R Zhang W Naseem M Puccini A Berger MD Soni S. Cxcl9, CXCL10, CXCL11/CXCR3 Axis for Immune Activation - A Target for Novel Cancer Therapy. Cancer Treat Rev (2018) 63:40–7. 10.1016/j.ctrv.2017.11.007 PMC5801162 29207310

[111] Jin BY Campbell TE Draper LM Stevanović S Weissbrich B Yu Z. Engineered T Cells Targeting E7 Mediate Regression of Human Papillomavirus Cancers in a Murine Model. JCI Insight (2018) 3:5715. 10.1172/jci.insight.99488 PMC5931134 29669936

[112] Waldman AD Fritz JM Lenardo MJ. A Guide to Cancer Immunotherapy: From T Cell Basic Science to Clinical Practice. Nat Rev Immunol (2020) 3:250. 10.1038/s41577-020-0306-5 PMC7238960 32433532

[113] Ott PA Hu Z Keskin DB Shukla SA Sun J Bozym DJ. An Immunogenic Personal Neoantigen Vaccine for Patients With Melanoma. Nature (2017) 547:217–21. 10.1038/nature22991 PMC5577644 28678778

[114] Sahin U Derhovanessian E Miller M Kloke B-P Simon P Löwer M. Personalized RNA Mutanome Vaccines Mobilize Poly-Specific Therapeutic Immunity Against Cancer. Nature (2017) 547:222–6. 10.1038/nature23003 28678784

[115] Alspach E Lussier DM Miceli AP Kizhvatov I DuPage M Luoma AM. Mhc-II Neoantigens Shape Tumour Immunity and Response to Immunotherapy. Nature (2019) 574:696–701. 10.1038/s41586-019-1671-8 31645760 PMC6858572

[116] Castle JC Kreiter S Diekmann J Lower M van de Roemer N de Graaf J. Exploiting the Mutanome for Tumor Vaccination. Cancer Res (2012) 72:1081–91. 10.1158/0008-5472.CAN-11-3722 22237626

[117] Gubin MM Zhang X Schuster H Caron E Ward JP Noguchi T. Checkpoint Blockade Cancer Immunotherapy Targets Tumour-Specific Mutant Antigens. Nature (2014) 515:577–81. 10.1038/nature13988 PMC4279952 25428507

[118] Khanolkar A Badovinac VP Harty JT. CD8 T Cell Memory Development: CD4 T Cell Help Is Appreciated. Immunol Res (2007) 39:94–104. 10.1007/s12026-007-0081-4 17917058

[119] Kreiter S Vormehr M van de Roemer N Diken M Löwer M Diekmann J. Mutant MHC Class II Epitopes Drive Therapeutic Immune Responses to Cancer. Nature (2015) 520:692–6. 10.1038/nature14426 PMC4838069 25901682

[120] Kumai T Lee S Cho H-I Sultan H Kobayashi H Harabuchi Y. Optimization of Peptide Vaccines to Induce Robust Antitumor Cd4 T-Cell Responses. Cancer Immunol Res (2017) 5:72–83. 10.1158/2326-6066.CIR-16-0194 27941004 PMC5221568

[121] Melief CJM van der Burg SH. Immunotherapy of Established (Pre)Malignant Disease by Synthetic Long Peptide Vaccines. Nat Rev Cancer (2008) 8:351–60. 10.1038/nrc2373 18418403

[122] Kluger HM Tawbi HA Ascierto ML Bowden M Callahan MK Cha E. Defining Tumor Resistance to PD-1 Pathway Blockade: Recommendations From the First Meeting of the SITC Immunotherapy Resistance Taskforce. J Immunother Cancer (2020) 8:e000398. 10.1136/jitc-2019-000398 32238470 PMC7174063

[123] Mukherjee S. The Emperor of All Maladies: A Biography of Cancer. Scribner (2010).

[124] Fairfax BP Taylor CA Watson RA Nassiri I Danielli S Fang H. Peripheral CD8+ T Cell Characteristics Associated With Durable Responses to Immune Checkpoint Blockade in Patients With Metastatic Melanoma. Nat Med (2020) 26:193–9. 10.1038/s41591-019-0734-6 PMC7611047 32042196

[125] Valpione S Galvani E Tweedy J Mundra PA Banyard A Middlehurst P. Immune-Awakening Revealed by Peripheral T Cell Dynamics After One Cycle of Immunotherapy. Nat Cancer (2020) 1:210–21. 10.1038/s43018-019-0022-x PMC7046489 32110781

